# Tumor-to-Tumor Metastasis of Renal Cell Carcinoma to a Follicular Variant of Papillary Thyroid Carcinoma: A Case Report and Literature Review

**DOI:** 10.7759/cureus.23742

**Published:** 2022-04-01

**Authors:** Fatima Badawi, Abdelrazak Meliti

**Affiliations:** 1 Department of Pathology and Laboratory Medicine, King Abdulaziz Medical City, Jeddah, SAU; 2 Department of Pathology and Laboratory Medicine, King Faisal Specialist Hospital and Research Centre, Jeddah, SAU

**Keywords:** immunohistochemistry, thyroid neoplasms, tumor-to-tumor metastasis, follicular variant of papillary thyroid carcinoma, renal cell carcinoma (rcc), clear cell renal carcinoma

## Abstract

Renal cell carcinoma (RCC) is the most common renal malignancy. It has a variable clinical course with metastasis to unusual sites occurring months to years after the initial diagnosis. However, metastasis can also be the first presentation of RCC. Although relatively uncommon, the thyroid gland is the most common location for RCC metastasis in the head and neck region. Tumor-to-tumor metastasis is an exceedingly rare occurrence. Only 10 cases were reported of RCC metastasis to primary thyroid neoplasms.

We present a case of clear cell RCC metastasizing to a follicular of variant papillary thyroid carcinoma (FVPTC) 14 years after the initial diagnosis of RCC. A review of similar reported cases revealed that the most common primary thyroid recipient of tumor-to-tumor metastasis of RCC was FVPTC. The rich lymphovascular network in FVPTC compared to other thyroid tumors, which may promote the deposition of metastatic tumor cells, might explain this predilection.

Careful review of the clinical and radiological findings and checking for any history of malignancy when examining thyroid nodules is important for guiding further studies. Performing a targeted panel of immunohistochemical stains for any suspicious areas is also essential for the diagnosis of such unusual cases.

## Introduction

Renal cell carcinoma (RCC) is the most common malignant neoplasm of the kidney, representing 90% of primary renal tumors and 3% of adult malignancies. It has an unpredictable clinical course, with metastases to unusual sites occurring months to many years after diagnosis and treatment. Metastatic RCC may also be the first clinical presentation. It frequently metastasizes to different organs, including the lung, liver, bone, and contralateral kidney. While much less common, the thyroid gland is the most likely recipient of RCC metastasis in the head and neck [1-3].

The thyroid gland is a highly vascular organ. However, it is an unlikely location for metastatic disease. RCC is the origin of more than half of clinically detectable metastasis to the thyroid gland [1,4].

Metastasis of one tumor into another is an exceedingly rare phenomenon [5,6]. Literature review reveals only 10 reported cases of RCC metastasizing to primary thyroid neoplasms [2-4,7-12]. We report a peculiar case of clear cell RCC (ccRCC) metastasizing to a follicular variant of papillary thyroid carcinoma (FVPTC).

## Case presentation

A 63-year-old lady had a history of recurrent RCC. She underwent left radical nephrectomy 14 years prior to her current presentation and was diagnosed with ccRCC. The patient followed up on an annual basis; the follow-up was uneventful. Approximately a year ago, a right multifocal renal mass was discovered, for which the patient underwent a right robotic nephrectomy. Histological examination revealed grade 1 ccRCC, stage pT1b.

The patient was on regular renal dialysis and was eligible for renal transplant. During the clinical workup, a multinodular thyroid goiter was discovered, and investigation with ultrasound (US) demonstrated heterogeneous bilateral nodules, with three predominant nodules on the right lobe of the thyroid, with the largest nodule measuring 1.9 cm. No suspicious lymph nodes were seen. The nodules were sampled by US-guided fine needle aspiration (FNA). The FNA findings were consistent with benign follicular nodules; however, one of the nodules showed atypia of undetermined significance. The patient underwent total thyroidectomy at an outside facility and was diagnosed with follicular adenoma and background nodular goiter. The material was reviewed at our lab as part of her pre-transplant workup.

**Figure 1:**
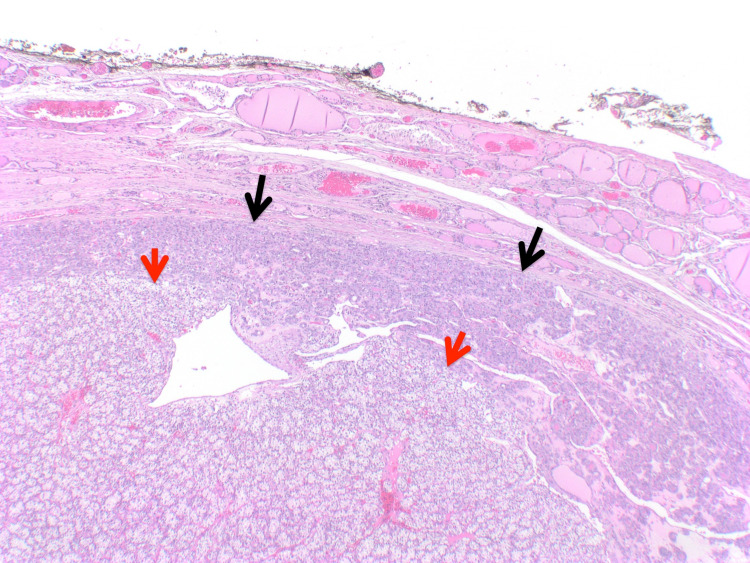
Largest thyroid nodule shows an encapsulated lesion composed of small follicles (black arrows), with a second population of clear cells (red arrows), hematoxylin and eosin stained section (H&E; x2).

**Figure 2:**
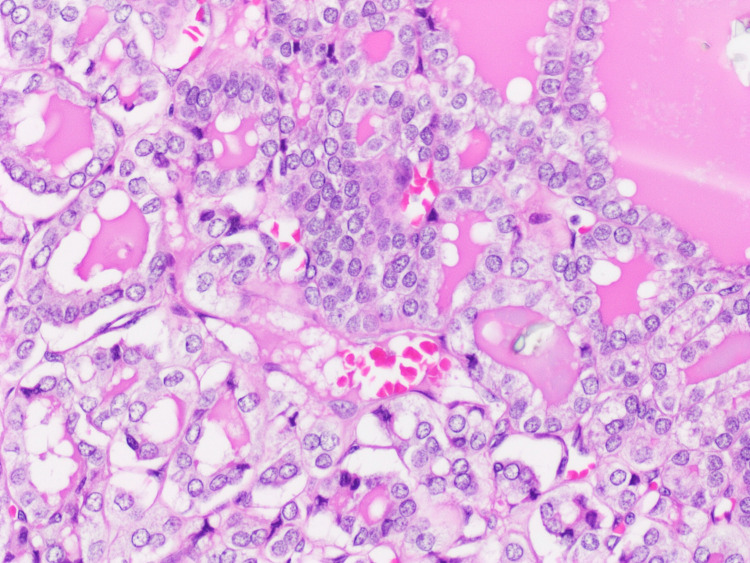
The lining cells exhibit nuclear enlargement, clearing, overlapping, and nuclear grooves, hematoxylin and eosin stained section (H&E; x20).

**Figure 3:**
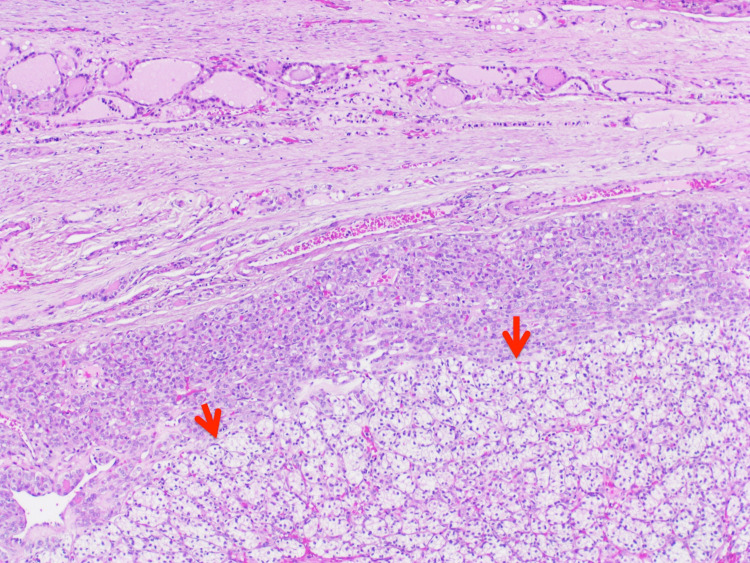
A second population of clear cells is seen adjacent to the follicles (red arrows), hematoxylin and eosin stained section (H&E; x4).

**Figure 4:**
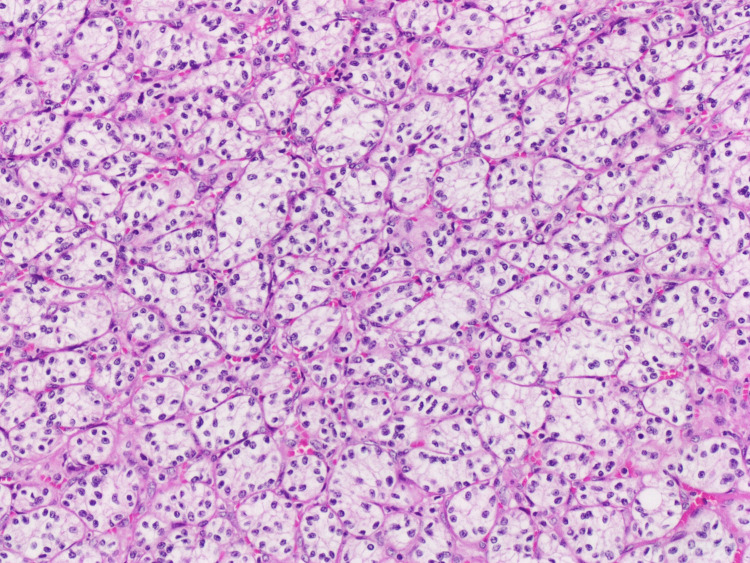
Clear cells are arranged in nests and sheets with small round nuclei, inconspicuous nucleoli, and a rich vascular background, hematoxylin and eosin stained section (H&E; x10).

Histologic examination of the largest right thyroid nodule revealed a well-defined encapsulated lesion composed exclusively of small, relatively uniform follicles. The lining cells exhibited nuclear enlargement, elongation, overlapping, clearing, and margination of chromatin, along with nuclear grooves (Figures 1, 2). A focal capsular invasion was evident. Juxtaposed to the follicular lesion, a second population of a slightly different neoplastic process was identified (1.6 cm). It was primarily composed of sheets and nests of cells with clear cytoplasm and a rich vascular network in the background (Figures 3, 4). The cells showed small round nuclei with inconspicuous nucleoli. No necrosis, mitoses, or vascular invasion were appreciated. Other nodules in the right lobe and the isthmus with pure morphology of papillary thyroid carcinoma were also appreciated. The remaining thyroid tissue showed multinodular goiter. The tumor was less than 0.1 cm from the surgical margin.

**Figure 5:**
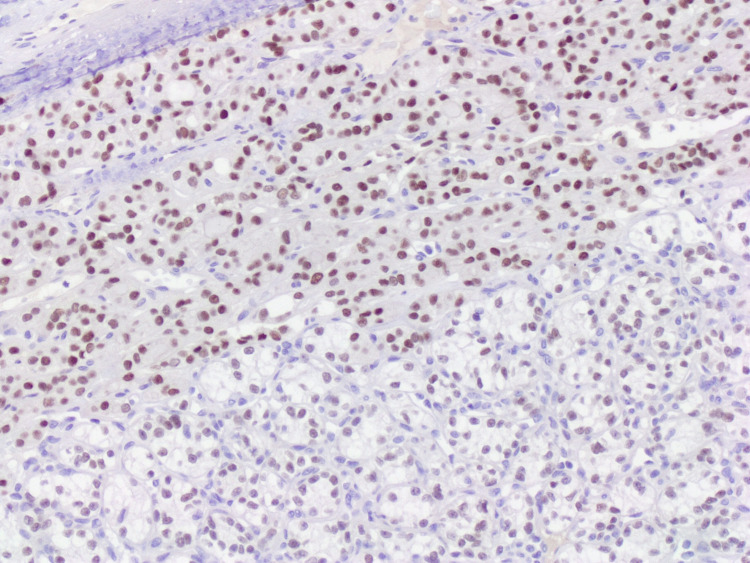
Both components were positive for PAX-8 (x10).

**Figure 6:**
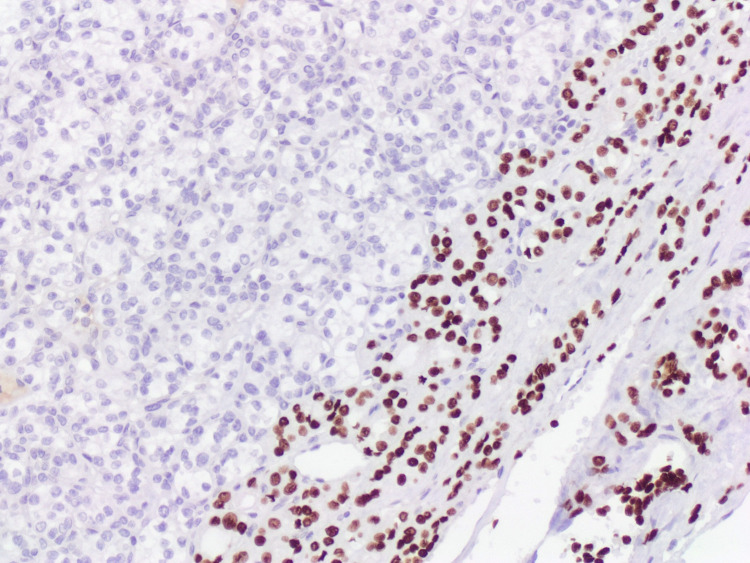
The follicular component was immunoreactive for TTF-1 (x10).

**Figure 7:**
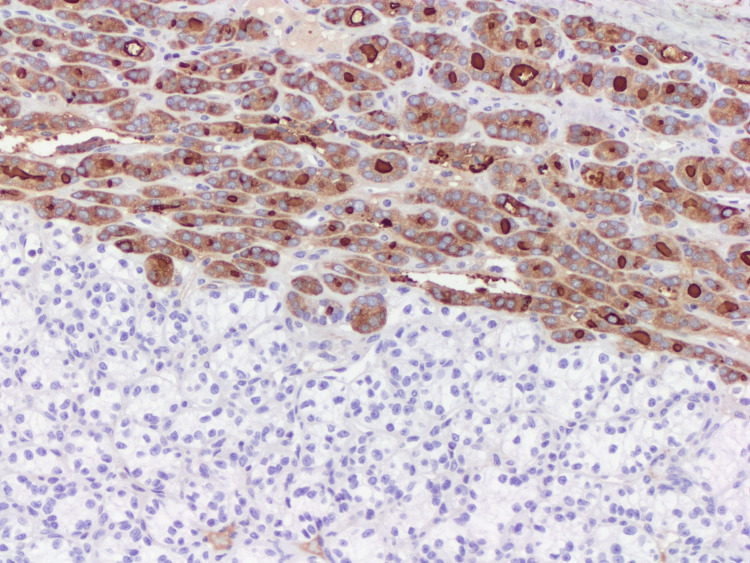
The follicular component shows immunoreactivity for thyroglobulin (x10).

**Figure 8:**
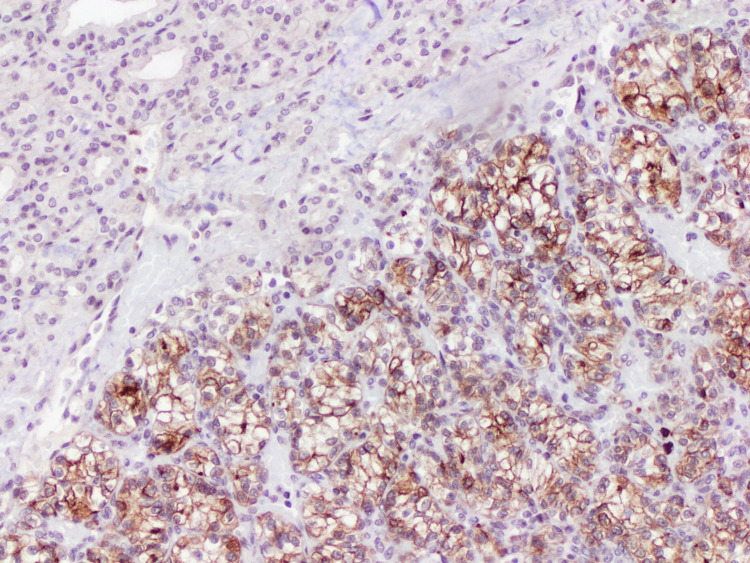
Membranous staining for CD10 in the clear cell component (x10).

Immunohistochemical (IHC) studies were performed. Both neoplasms were positive for PAX-8. The follicular component was immunoreactive to TTF-1, thyroglobulin, CK7, CK19, and HBME-1. In contrast, the clear cell component showed immunopositivity for CD10, carbonic anhydrase 9 (CAIX), galectin-3, and vimentin (Figures 5-8). The histomorphological features and the immunoprofile were consistent with tumor-to-tumor metastasis of ccRCC into papillary thyroid carcinoma, encapsulated follicular variant (FVPTC).

The patient underwent re-excision of thyroid tissue remnants three months later, which yielded multiple foci of papillary thyroid carcinoma. The largest focus was 0.2 cm in the greatest dimension.

## Discussion

There have been fewer than a dozen cases of tumor-to-tumor metastasis of RCC to thyroid neoplasms reported in the English literature (Table 1). Notably, RCC is the most frequent donor tumor metastasizing to thyroid neoplasms [4].

**Table 1:** Summary of reported cases of RCC metastasis to thyroid gland tumors, including the type of recipient tumor, interval from nephrectomy, other sites of metastases, and IHC profile of metastatic RCC. IHC, immunohistochemistry; FA, follicular adenoma; FVPTC, follicular variant of papillary thyroid carcinoma; M, months; PTC, papillary thyroid carcinoma; RCC, renal cell carcinoma; TG, thyroglobulin; Y, years

#	Author	Recipient Tumor	Interval	Other Metastasis	IHC of Metastatic RCC
1	Rosai (cited by Ryška and Čáp [7])	FA	NA	NA	TG (-)
2	Baloch and LiVolsi [8]	FVPTC	2Y	Liver, pancreas	TG, CK19 (-)
3	Wolf et al. [9]	FA	2Y	NA	NA
4	Ryška and Čáp [7]	Hurthle cell (oncocytic) carcinoma	13M	Subcutaneous	AE1/AE3, vimentin, EMA (+); TG, CEA, CK19, calcitonin (-)
5	Qian et al. [10]	Hurthle cell Adenoma	synchronous	None	Vimentin, CD10 (+); TG, TTF-1 (-)
6	Koo et al. [3]	FA	5Y	None	CK, CD10, galectin-3 (equivocal); TG, TTF-1, calcitonin (-)
7	Bohn et al. [11]	PTC	2Y	Spinal vertebrae	RCC (+); TG (-)
8	Yu et al. [12]	FVPTC	3Y	None	CAM 5.2, CD10, vimentin (+); TG, TTF-1, RCC (-)
9	Medas et al. [2]	FA	6Y	None	CD10 (+); TG, TTF-1, galectin-3 (-)
10	Kefeli and Mete [4]	FVPTC	18Y	None	PAX-8, CD10 (+); vimentin (equivocal); TG, TTF-1, CEA, calcitonin (-)
11	Current case	FVPTC	14Y	Contralateral kidney	PAX-8, CAIX, galectin-3, vimentin (+); TG, TTF-1, CK7, CK19 (-)

Tumor-to-tumor metastasis has been defined using strict criteria by Dobbing and by Campbell et al.: the presence of two or more distinct tumors, the recipient tumor must be a true neoplasm, and there must be a true metastatic deposit within the neoplastic tissue of the recipient tumor. These criteria exclude collision tumors, lymphovascular spread into another tumor without actual invasion, and metastasis into hematopoietic malignancies [5,6,8,12].

Metastasis to the thyroid gland is thought to occur by hematogenous spread through the paravertebral veins [1]. However, the pathogenesis of tumor to tumor metastasis is poorly understood. Two proposed hypotheses are the mechanical and metabolic (seed and soil) hypotheses. The mechanical hypothesis suggests that metastatic tumor cells reach the recipient tumor due to rich vascular supply compared to non-tumorous tissue, whereas the metabolic hypothesis postulates that recipient tumors provide a fertile “soil” for donor tumor cell “seeds” to proliferate [3,12].

FVPTC appears to be the most common recipient thyroid neoplasm for tumor-to-tumor metastasis. It has been demonstrated to have a dense lymphovascular network compared to other primary thyroid tumors. That may promote the deposition of metastatic tumor cells, in keeping with the mechanical hypothesis [3,8,12].

Diagnosing RCC metastasis to thyroid tumors solely on routine H&E examination can be a very challenging task. RCC metastasis commonly presents as a solitary nodule, clinically and radiologically mimicking primary thyroid disease. RCC exhibits an unpredictable behavior with the possibility of metastasis preceding or up to 26 years post-diagnosis of RCC [7,10]. In addition, metastatic ccRCC can mimic other thyroid diseases with clear cell changes, such as primary thyroid tumors with clear cell features, intrathyroidal parathyroid tumors, and paraganglioma [4]. A history of RCC or nephrectomy should raise the suspicion of metastatic carcinoma.

Morphological findings suggestive of metastatic RCC include clear cell morphology, abundant cytoplasmic glycogen and fat, hemorrhagic glandular spaces, sinusoidal-type vascular network, and multiple tumor nodules [4,13]. Facing some of these unusual features in a thyroid tumor should prompt pathologists to perform further studies to exclude metastatic RCC.

## Conclusions

Tumor-to-tumor metastasis is an extremely rare occurrence. Being aware of the clinical and radiological findings and any remote history of malignancy while examining thyroid tumors is crucial. Performing appropriate IHC studies on suspicious cases is a key to reaching the correct diagnosis and, subsequently, providing the appropriate management for the patient.
